# A Spinal Root of the Optic Nerve

**Published:** 1881-02

**Authors:** 


### A SPINAL ROOT OF THE OPTIC NERVE.

Stilling, of Strasburg, showed preparations to the International Ophthalmological Congress at Mailand, in September last, which he believes demonstrate the existence of a spinal root of the optic nerve, which brings the retina into direct connection with the medulla. This root passes from the external corpus geniculatum, in a winding course, deep between the bundles of the crus cerebri, and can be traced into the pons; and it appears to course down in the direction of the medulla, although its further progress cannot be demonstrated. The existence of this branch is interesting on account of the light it throws on certain physiological relations between the medulla and the retinæ, and may constitute the hitherto undiscovered link between certain diseases of the spinal cord and of the optic nerve.

